# Supplementary figures and images for: Evidence of stage progression in a novel, validated fluorescence-navigated and microsurgical-assisted secondary lymphedema rodent model
Source: PLoS One. 2020 Jul 23;15(7):e0235965. doi: 10.1371/journal.pone.0235965 (PMC7377415; doi:10.1371/journal.pone.0235965)

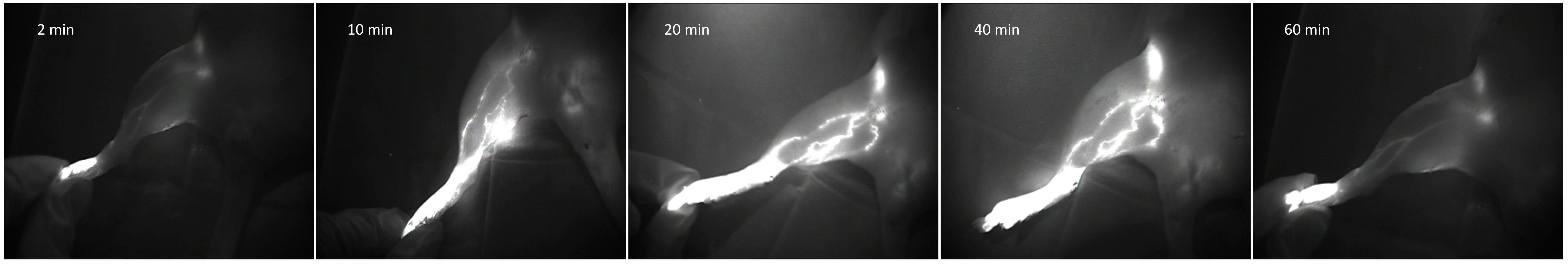

Supplement: S1 Fig — During preoperative mapping of the lymphatic system of the rat hind limbs and the intraoperative real-time navigation, an early appearance (2 min) of ICG contrast in the lymphatics was noticed by NIR visualization with the Fluobeam® camera. A clear contrast of the lymphatics of healthy limbs was detected with NIR visualization until 1 h after injection. By then most of the ICG was cleared from the limbs. However, at the subcutaneous injection sites, the ICG signal remained almost unaltered. This is probably to be related to the local binding of ICG molecules to tissue proteins. For further analysis, the injection sites were therefore not included in the region of interest (ROI). (TIF) [file pone.0235965.s001.tif]
