# Supplementary material for: Evidence of stage progression in a novel, validated fluorescence-navigated and microsurgical-assisted secondary lymphedema rodent model
Source: PLoS One. 2020 Jul 23;15(7):e0235965. doi: 10.1371/journal.pone.0235965 (PMC7377415; doi:10.1371/journal.pone.0235965)
Supplement: Attachment — Submitted filename: Response to Reviewers_Plos One.docx [file pone.0235965.s002.docx]

**Dear**

**Prof. Raffaele Serra, M.D., Ph.D
Academic Editor
PLOS ONE**

We gratefully thank you for your editorial work, the comments and the opportunity to improve our manuscript based on all suggestions. As requested, we hereby submit a rebuttal letter with detailed explanations of the concerns raised during the review process. The suggestions and comments for improvement of the reviewer were taken into account and incorporated into the revised version of the manuscript, which has been significantly improved. Many issues raised by the reviewer were discussed in great detail, most of the advices taken into account, and the reference list expanded with the suggestions made. However, the rebuttal letter and subsequent discussion also upholds that sometimes the definition on scientific originality and conception of an ideal animal model might be subject of personal interpretation and the scientific question addressed, rather than tangible substantiation. We hope to have successfully put the paper into an optimized perspective in terms of readability and understanding for the readership, taking into account that the modifications and new references slightly increased the length of the manuscript.

Thank you once again.

Best regards,

The authors

**Response to reviewers**

Reviewer #1: In this paper, the authors describe results from a rat lymphedema model in which lymphatic vessels and nodes are removed and the resulting limb volume changes and changes within the skin are described. The observed pathology (namely fibrosis and adipocyte expansion), the methods for quantifying changes in function with ICG, or the animal model itself are all variations of work reported by others. In the authors attempt to establish the novelty of the findings, many of the claims are grossly overstated and overlook or over-simplify a significant body of literature regarding mechanisms of lymphedema development in animal models. In addition, the manuscript is missing the appropriate statistical analysis to draw conclusions. The authors need to clearly focus on the limitations that this model addresses, the novelty of the findings themselves, and the value the model adds to the field. Specifics of these are as follows:

**A: We appreciate the reviewers’ comments and its careful review of our manuscript. The presented and validated animal model has never been published before by others. We agree with the reviewer that the presented model is further developed based on preceding studies and pre-published literature regarding the SL development in animal models. These preexisting works have been cited carefully and referred to in the manuscript. The exclusive novelty of the findings in the presented model involves stable long-term extremity SL in the hind limb. This was additionally achieved without radiation, a fact that is critical for the evaluation of the inflammatory and fibrotic pathogenesis, as radiation per se causes these processes independently of lymphedema disease. We agree with the reviewer that some of the methods applied for the validation of our model have been described and published before, while some have been exclusively applied in our study (microsurgical guidance of dissection to improve surgical accuracy and ICG lymphography before, during, and after the induction of the model as well for surgical navigation). We aimed at optimizing the preexisting models and validate the incorporated refinements using deliberately an already applied methodology in order to offer a real and comparable alternative to the preexisting versions for further investigations. In our opinion, it would be a mistake to disregard the preexisting published methods and establish a completely novel model or SL outcome assessment just to offer a novel methodological description. Therefore, we put special emphasis on employing translational relevant criteria, methods and outcome validation. Lymphedema models that chiefly focus on a particular mechanistic pathway but lose the translational nexus might be of great scientific relevance, but if not encased correctly are endangered of being inconsequential. Lymphedema is a disease affecting patients on a daily basis, as such, the primary focus of the SL models should be to safeguard a translational capacity.**

**In addition, we appreciate the specifics of these comments which we are going to reply to as follows and incorporate into the revised manuscript wherever possible or meaningful in order to preserve the originality of the manuscript.**

1) Lipid accumulation and fibrosis by collagen deposition in the skin, while certainly necessary components of pathology in lymphedema models, has been reported in many other animal models (some of which have been cited) and some which are missing: (Swartz, J Biomech, 1999; Mendez, AJP, 2012; Savetsky, AJP, 2014).

**A: We gratefully thank the author for providing some further references for our paper. As the reviewer considered it to be important to cite all the publications in this regard, we gladly added the suggestions to the references.**

The authors claim “Moreover, for the first-time SL stage migration was validated by characteristic histological alterations, e.g. subdermal mast cell infiltration, adipose tissue deposition, and fibrosis by increased skin collagen expression.” While the term stage migration is not used in the non-clinical lymphedema literature (including reference 12 which the authors cite on line 54 to establish the term), many of the papers by the research groups of Melody Swartz, Babak Mehrara, Jeremy Goldman, Brandon Dixon, Michael Detmar, Stan Rockson, etc.. report events that describe the key features of this process in animal models. It is an overstatement to suggest the observations in this model have not been made before.

**A: Thank you for your literature review, we have taken over some papers of the working group to support and underline preceding research work on this issue as suggested. Intentionally, we have introduced the term stage migration for our preclinical lymphedema model, since it is well-defined term in the clinical literature and necessary in terms of translational research (reference 12= description of clinical disease stages and their modification by the international society). We have added a definition to the methods section for clarification: “Stage migration involves histopathological dermal changes, local cellular alterations, and modification of the connective tissue, consecutive to interstitial progressive fluid accumulation”. We intend to identify stage migration as a process and hereby a conjunction of several histological, histomorphological and functional alterations over time. Maybe we failed to deliver this concept during the prior version of the manuscript. Consequently, the revised manuscript includes some relevant modifications and put this issue into a new, better readable perspective**

Another example “Stage migration is a key feature of the disease but, upon now, it has never been reported in an animal model as a consistent process.” (line 87). In their effort to oversell the model, the importance of the novelty of the model and what it could be used for is lost.

**A: We accept the reviews concerns and have revised the paragraph. The revision includes the designation of preceding research and involves the statement that it has not been described as a consistent process. We did not intend or put any effort to oversell the model. Nevertheless, to improve comprehension and readability, the reader should indeed be focused on the novelty of our model, which we hope you are going to share after reading our revision.**

2) In addition, animal models of lymphedema have been crucial in understanding many of the molecular mechanisms important in lymphedema development, a few of which have been cited but many which are missing: (Nores, Nat Comm, 2018; Nores, J Inv Det, 2017; Tian, Sci Trans Med, 2017; Ogata, J Inv Det, 2016; Gousopoulos, J Inv Det, 2017) which have also provided therapeutic targets that have been effective in the animal models, albeit their clinical effectiveness remains to be established (Gardenier, Nat Comm, 2017; Tian, Sci Trans Med, 2017; Honkonen, Ann Surg, 2013).

**A: We thank the reviewer for the valuable comments and his precise review of the literature. Most of the suggested references have been added to the revised manuscript, when relevant for the context of the paper, while some have already been included in the first version.**

The paper here provides no new mechanistic insight or evidence that the model can address the shortcomings of these other models in yielding new mechanistic insight and statements like “and the molecular key players of stage migration have not been yet identified” (line 63) are incorrect and give the impression that the authors are unaware of the work in the field that sets the context for their animal model.

**A: We thank the reviewer for raising his concerns. Indeed, we believe that this paper provides new mechanistic insights and do not share the unilateral opinion reflected in this comment. A special subpopulation of lymphedema associated fibroblasts have been found in correlation to fibrogenesis and adipogenesis over time. As described in the manuscript, the main focus was to establish a hind limb model with clear definition of lymphedema, long term stability (chronification / stage migration as a process), and a translational validation. In addition, we aimed at reducing confounding methodological variables like indiscriminate soft tissue resection, high complication rates, and the currently accepted view that hind limb models might only be generated with the addition of radiotherapy during the surgical induction. When we intended to study therapeutic interventions, it seemed unmanageable to us to trade unspecific cell and tissue actinic alterations in order to get a lymphedema.**

**Nevertheless, we have revised the quoted sentence and have put it into a new perspective in the revised version: “Currently, drugs or cellular therapy do not play a therapeutic role, since the effects of cellular pathogenesis, immunological regulatory mechanisms, and the molecular key players of stage migration have only been identified to a limited degree.”**

**The necessity to put light on the insight into the mechanistic development and regulation of the disease is a major focus. However, conceding translational correctness is not in the intention of medical science. Despite many excellent publications cited by the reviewer, current treatment strategies are merely conservative or surgical (ablative and supermicrosurgical). The unique curative approach for some patients is vascularized lymph node transfers and lymphovenous anastomoses. These procedures have been shown by recent systematic reviews to significantly improve the patients outcomes (B Mehrara cancers 2020) yet are limited to a few highly specialized centers. We do perform them regularly, but miss preventive or adjuvant drugs or immunotherapeutic agents. Sadly, despite many years of mechanistic insight (especially in the tail models) we lack such opportunities for our patients. Further, of 339 trails registered in clinicaltrails.gov for secondary lymphedema only 4 were focused on novel drugs (QBX258, Ubenimex, Pazopanib, and Lymfactin). We understand this as an assignment for all in the field to further develop novel perspectives and optimized models.**

**This opinion is shared and discussed by other authors. As an example: The latest consensus document of the international society of lymphology (Lymphology, 2016), the official international society for the study and treatment of lymphedema, share our point of view with statements like** “Research in molecular lymphology including lymphatic system genomics, proteomics, and “systemomics” should be greatly expanded. With the cellular and molecular basis of lymphedema-associated syndromes better defined, an array of specific biologically-based treatments including modulators of lymphatic growth and function should become available. Improved imaging techniques and physiological tests need to be devised to allow more precise non-invasive methods to measure lymph flow dynamics and lymphangion activity.” **And regarding drug therapy** “The injection of hyaluronidase or similar agents to loosen the extracellular matrix is of unclear benefit and may actually be harmful….Efficacy of boosting immunity by intraarterial injection of autologous lymphocytes is unclear and needs independent, reproducible evidence…Recent proposals for the use of anti-inflammatory pharmaceuticals have not yet demonstrated efficacy and may face drawbacks if administered long-term.”, **and they added regarding preclinical tissue engineering** “The implantation of tubes to transport lymph or engineered tubes/devices to promote new substitute lymphatic growth have not yet documented long-term value in large studies, and these techniques are continuing to undergo investigation.”. **It is plausible to conclude, that more basic research and divergent animal models with translational focus might be beneficial for the field and patients.**

3) NIR imaging has been used both to access lymphatic function in lymphedema models (Gousopoulos, Am J Path, 2016; Kwon, PLoS One, 2014; Nores, Nat Comm, 2018) and to guide lymphatic injury in creating animal models of lymphedema (Weiler, Sci Rep, 2019). The authors claim on line 79 “… yet it has not been applied so far for in-vivo studies” is incorrect.

**A: We thank the reviewer for its valuable comment and agree that we have to put the paragraph into a new context: ICG bases NIR imaging has been used both to evaluate lymphatic function in animal models and NIR functional imaging [20,28–30], nevertheless it has not been used a standardized tool for pre- and intraoperative lymphangiographic navigation nor to guide a precise lymphatic injury for *in-vivo* studies. This modification was included in the new version of the manuscript.**

4) The statement in the abstract “In contrast to the few models reported so far, in this study the authors decidedly avoided the use of radiation for the lymphedema induction” is a significant overstatement and generalization of the lymphedema literature given all of the same papers mentioned above. In addition, many animal models of lymphedema have been created without radiation (above papers) and have shown to be made more severe with radiation (Lynch, AJP, 2015; Avraham, AJP, 2010).

**A: We disagree with the comment, as the purpose of this statement is to elucidate our concept. A divergent point of view should not be regarded as a unilateral statement or overstatement. We agree, that a few limb models (Wang 85 and Kandel 90) have been proposed without radiation but raise several limitations discussed in the manuscript and summarized in Table 1: unclear lymphedema, no long-term stability of SL in hind limb, and prohibitive complication rates. The models cited by the reviewer as example of radiation-free models should not be compared to extremity models as these are tail models. They are all generated by resection of soft tissue with no direct sight on the lymphatics, no intraoperative lymphography, and no resection of lymph nodes. This last argument is another crucial translational shortcoming of the tail models since most of the oncological SL cases happen to patients after lymph node dissections. Also, considering that lymph nodes drain a specific lymphosomes and are locally the main station of immunoregulation, models without lymph node resection might miss to replicate immunopathogenic changes of lymphedema and fail to reproduce an immune-dysbalanced state associated with higher locoregional cancers and hr soft tissue infection rates. Finally, the tail models have different histological composition and lack the opportunity of a direct comparison to the unaffected extremity in terms of translational research. Other limitations of tail models are discussed below and, in the manuscript.**

5) In the statistical analysis section the authors state that they used a one-way ANOVA. While this tells you that the means are different, it does not make any claims as to which means are different from one another. Also it is not clear from the figures, when any of these tests were actually applied and what the results of the test are. If making multiple comparisons (as should be done in Fig 2, 3, and 5) a a post-hoc correction must be applied. The same is true for the Wilcoxon signed-rank test. Furthermore, since the appropriate statistical test is often different for each figure, the test used to determine significance for each figure should be clearly stated.

**A: ANOVA involves calculating variances by linear regression under the assumption for H0 that all means are equal and if rejected, as correctly stated by the reviewer, implies that the means are different. As underlined by the reviewer, one-way ANOVA tells you essentially nothing about what groups observed are different from another. Here multiple comparisons are necessary and, of course, were applied during this project. In order to keep the statistic description clean and simple for the readership of PLOS One, we did not include to many details nor all exact test applied in the methods section and in each figure legend. We cannot remember any publication of this journal or others, related to the topic, where this level of detail was included. However, we do completely agree with the reviewer that accuracy in statistical methods is of crucial importance and sadly is incomplete or imprecise in many papers of different biomedical disciplines. Interestingly, not many models of the cited by the reviewer performed a long term follow up of most of the outcomes to the extend we did and subscribed to this level of statistical precision. Since one of the major aims of this project is to validate the model to the highest possible scientific standards, we kindly thank you for the opportunity to prove that in this matter we proceeded rigorously. For this revision we also re-examined the results with faculty of the statistics department at the Heidelberg University, using another software (SPSS). As expected, the results obtained with SPSS and PRISM 8.0 were identical.**

**The complete methodological explanation of the statistical analysis follows here and was added, in a reduced extend, to the revised manuscript and figure legend. If the detail and length outgrow the expectations of the journal, we kindly ask the editor to let us know what to exclude for publication.**

**The dataset for the volumetric hind limb difference measured by plethysmography (water displacement) was tested for normal distribution with Prism 8.0 using the Shapiro-Wilk test with Alpha 0,05. Once the data passed the test, first one-way ANOVA was tested in order to determine an overall statistical difference of the two paired groups (intervened and control limb). The results were corrected with Bartlett’s test and proved to be significant for alpha 0,05 and alpha 0,01. Further multiple comparisons between the different time points were performed by 2-ways ANOVA and the assumption of equal variability tested with Geisser-Greenhouse correction. ANOVA tests at Prism 8.0 automatically includes the Sidak post-hoc test in the multiple comparisons test. For additional validity, the multiple comparisons were repeated with the Bonferoni and Benjamin, Krieger and Yekutieli post-hoc tests with no statistical differences from the Sidak results. Although it has less statistical power, the dataset was also tested again by multiple t-test per row with individual p values and false discovery rate with the two-stage step-up method of Benjamini, Krieger and Yekutieli as well as statistical significance correction by Holm-Sidak for multiple comparisons with alpha 0,01. Here the statistical significance was in concordance with the results of the multiple comparisons by 2-ways ANOVA.**

**The dataset for the ICG clearance capacity did not pass the D’Angostino & Pearsons nor the Shapiro-Wilk normality test with alpha 0,05. Therefore, the Kruskal-Wallis test with Friedman correction was applied with alpha 0,05 and 0,01. Multiple comparisons were included using the two-stage step-up method of Benjamini, Krieger and Yekutieli and alpha 0,05 and 0,01.**

**The statistical analysis of the mean fluorescence intensity of PSR stained collagen was performed with one-way ANOVA and corrected with Barlett’s test at α 0.05, since the dataset distribution proved to be normally distributed by the Shapiro-Wilk test (α 0.05). For a better understanding, the statistical tests used were added to the respective figure legends.**

**(During the revision we realized that the wrong test was described in the method section of the first manuscript version. Wilcoxon signed-rank test was never used since more than two time points or groups were analyzed for ICC. Here the more accurate test for non- parametric data is Kruskal-Wallis. We apologize for making the wrong description in the first place)**

6) The figures are very poor resolution and some of the labels and fonts are difficult to read.

**A: On the contrary, the original images are in high resolution and the Tiff files frequently exceeded several times the 20 MB limit of PLOS One. With Photoshop the images were therefore converted to jpeg but the resolution remained good. Individually after uploaded the quality remained fine, however during the automatic conversion of the manuscript we also noticed that in the Pdf file the images had lower resolution. At that moment we believe that it is normal and desired by the journal to reduce size of the files by decreasing the resolution (for example for better distribution to the reviewers). For the publication or revision, we will gladly provide the images in full resolution or could discuss a practical solution with the technical department of PLOS One.**

7) Measuring fluorescence at a single time point to quantify lymphatic function could produce erroneous results as the clearance of tracer by lymphatics has been shown to be exponential (Karlsen, AJP, 2012; Karlsen, ATCB, 2018). In addition, repeat injections of ICG has been shown to be problematic in inducing an inflammatory response (Weiler, Front Phys, 2013) additionally raw ICG fluorescence is highly dependent on protein binding (Weiler, J Biom Opt, 2012) and thus differences in fluorescent intensity (Fig 3) are difficult to interpret.

**A: We are grateful to the reviewer for the technical input in this comment. The single time point (1h) has been defined during the establishment of the model by real-timing imaging, since 1 hour after the subcutaneous injection the signal intensity was almost completely cleared of the hind limbs. We have added this information to the method section. From a translational perspective, it is also the timepoint recommended for lymphangiographic diagnostic imaging on patients with SL.**

**Until now we have not observed any inflammatory response in the applied ICG concentrations in our setting or in in-vitro tests on representative skin cell subpopulations (unpublished own data). Yet we coincide that is an issue in higher concentrations. We also agree to the reviewer that raw ICG fluorescence is dependent on protein binding. If the editor considers these aspects imperative for the readership, we would discuss these topics in the respective section. In our opinion, these and other aspects are good examples why standardized and repetitive protocols are necessary and have been applied.**

8) The water displacement technique seems like it could be highly subjective to error as the method drastically depends on how far one submerges the limb into the container. The authors have done their best to control for this but it would be helpful to have a few measurements of intraoperator and interoperator variability of a few blinded users to get a better appreciation of how accurate and repeatable the measurement is.

**A: We appreciated that the reviewer recognizes our effort in delivering methodological correctness on this translational parameter. In the clinical setting we witnessed the limitations mentioned by the reviewer. Fortunately, we reduced variability by applying strictly standardized plethysmography protocols on patients with lymphedema. This challenge was already considered during the study design. To this regard, all water displacement measurements were performed by the second author in a commitment to reduce the interoperator variability. This information was added to the method section. How the polypropylene container itself was adapted to avoid possible unintentional submersion of the limbs in the container, was already described in the method section of the prior manuscript. For better visualization, it was also marked in red in this corrected version.**

9) Normalizing the life span of a rat and a human to equate the temporal kinetics of lymphedema development (i.e. that 1 year equals 11.8 days) is not appropriate. The remodeling of tissue depends on the turnover rates of matrix constituents, the proliferation rates of cells, etc… and these do not scale linearly with life span. In addition the statement that “nearly all reported incidences of SL were reported in the first 3 years” is not correct. While most large clinical studies have stopped tracking patients after 36 months, those that have followed patients further out continue to observe new occurrences of lymphedema long after 36 months (Armer, Lymphology, 2010; Petrek, Cancer, 2001).

**A: We completely agree with the reviewer that the life span of rats cannot be directly linked to the life span of humans. Consequently, we already included the following sentence in the previously submitted version, and have now marked it red for better recognition: “The authors are aware that the chosen follow-up period in the rats, of 45 days, might not fully represent a translational equivalent of 3 human years. Temporal kinetics of extracellular matrix, cell proliferation, enzymatic turnover rates, and DNA repair might vary interspecies during aging. In order to minimize this error, the best available translational approximation currently available in the literature[33] was employed.” We humbly consider that a translational follow-up based on published life span estimations is scientifically better than an arbitrary time lapse (as in the other models). We still believe that this follow up period is the most suitable approach for interpreting the data. The objected sentence “nearly all reported incidences of SL were reported in the first 3 years” has been put into a new perspective in the revised version in concordance to the cited long-term studies.**

10) One of the papers’ points is that the tail model damages more than just the lymphatics and thus is not clinically equivalent to human lymphedema. Given that a large amount of our understanding of the molecular mechanism’s involved in lymphedema are from the mouse tail model, there would be value in alternative models that overcome its limitations in order to understand new biology. However, it appears that all of the results in the pathology in this surgical model in the rat are identical to what has been shown in the mouse tail – lipid accumulation, mast cell infiltration, fibrosis and collagen deposition, impaired ICG clearance, etc… It would be helpful if the authors could emphasize the data in this model that produces unique results over models whose limitation it is seeking to overcome.

**A: We are glad that the reviewer recognized in our model an alternative to the tail models and emphasized that it can reproduce hallmarks of lymphedema like lipid accumulation, mast cell infiltration, fibrosis, collagen deposition, and impaired ICG imaging. We also agree with the reviewer that the tails models have significantly contributed to the preexisting literature, as the reviewer correctly stated, and should be considered for further research in the field. Nevertheless, we believe in the necessity of an alternative model to the rodent tail models and are hereby thankful to the reviewer for the discussion he has started regarding the comparison of hind limb and tail SL models.**

**As stated in the manuscript, we do claim that it is important to keep the ongoing discussion regarding the lack of surgical meticulousness and intraoperative mapping inherent to the rodent tail models. Due to these shortcomings the researchers are likely to induce unspecific soft tissue edema or collateral venous insufficiency, in addition to a plausible lymphedema caused by injury of relevant lymphatic structures. Though, the rat tail in our opinion does not seem to anatomically, functionally, nor histologically be the optimal translational body segment compared to a human limb, which is mainly affected by SL. Since most of the lymphatic transport of humans is generated passively by muscular contraction of the extremities, the physiologic (and hereby pathophysiologic) lymphatic transport is replicated much more accurately in the limb models. Furthermore, extremity models provide a significant advantage in translational research as they involve the opportunity of a direct comparison to the unaffected extremity. Systemic vs local differences can be hereby explored in the same experimental subject under equal conditions. Also, this approach is likely to reduce the number of animals needed to answer a scientific question (reduce principle of animal experimentation).**

**As stated above, we do believe in the contribution and necessity of SL tail models. However, depending on the scientific hypothesis (for example immunopathogenesis of SL, cell therapy applications or evaluation of supermicrosurgical outcomes) the hind limbs are to us a better alternative. Further, it would be interesting that the mechanistic insights reveled in the tail experimentation could be confirmed on a translationally more “humanized” body segment and under more precise induction. The fact that the academic discussion started during this review about the models, a novel approach is likely to be also of great interest to the field. In the end, it is the scientific community and in particular the readership of PLOS One that should be able to choose the ideal rodent SL model (tail vs extremity and radiation vs no-radiation) to address their specific research questions.**
