# Supplementary material for: Evidence of stage progression in a novel, validated fluorescence-navigated and microsurgical-assisted secondary lymphedema rodent model
Source: PLoS One. 2020 Jul 23;15(7):e0235965. doi: 10.1371/journal.pone.0235965 (PMC7377415; doi:10.1371/journal.pone.0235965)
Supplement: Attachment — Submitted filename: Response to Reviewers II.docx [file pone.0235965.s003.docx]

**Response to the Revierer´s Comments Revision 2, PlosOne**

Reviewer #1: In this revised manuscript the authors have made some strong improvements including a detailed statistical methods section with the appropriate statistics applied and a broader inclusion of the relevant scientific literature to the into and discussion. There still are some major revisions that need to be made, some of which were brought up in the previous review and were not adequately addressed.

Major comments
1. I do not think it can be determined what exactly is being measured with the NIR imaging approach the way it was conducted here. As mentioned previously, when ICG is injected it binds to protein and there is a shift in the excitation and emission spectra and a gain in the quantum yield which has been shown to result in an increase in fluorescence of up to 500%. I would hypothesize that the total fluorescence intensity of an image in one of the operated rats taken at 1 hour after injection would be extremely brighter than an image taken immediately after injection. This is because the dye binds to protein before it is cleared by lymphatics, the intensity goes way up, and then the brighter ICG carried away where it leaks out further downstream. There was a lot of work done here and I am not asking that the NIR clearance studies be redone. However I do not think you can make definitive statements about these measurements relating quantitively to the clearance capacity of the lymphatics.

**A: We appreciate the reviewer’s clarification of the mechanisms of the ICG uptake and clearance, to which we fully agree and already agreed in the last response. Until now, only radioactive scintigraphy in a controlled setting and with a well-known radioisotope doses and kinetics is a truly quantitative imaging *in-vivo*. We followed a generalized trend “to quantify” a not strictly quantifiable fluorescence with the hope that the comparative control could compensate inexactitudes of the technical limitations, resulting in a semiquantitative analysis. Nevertheless, we agree that fluorescence “quantification” should not be considered as quantitative in strict terms. In the revised manuscript we have removed all parts where ICG clearance was described as quantitative analytics, and have replaced it by the term “semi-quantitative”.**

2. It is still not clear how the NIR images were quantified to produce the plots from the description in the methods: “Furthermore, the ICG fluorescence signal at one-hour post application of the gated extremities were gathered as counts per second (cps) by the VIS-NIR X-ray imaging optical device.” Usually in the context of fluorescence the term gating refers to setting a threshold for the intensity, below which the signal is considered 0. I think what the authors mean here is that they selected a ROI to include just the extremities and then calculated the fluorescence here. The authors also mentioned they validated the technique by imaging continuously for 1 hour in unoperated rats. I think this data would be helpful to include to provide a sense of how much dye is actually being cleared during this 1 hour time period. The statement that it is almost all gone contradicts several published reports that ICG can stick around for days at the **site of injection.**

**A: We appreciate the valuable comments: We have re-termed the area analyzed with the VIS-NIR X ray from “gated” to “region of interest (ROI)” as suggested and agree that is more adequate for the technique used. A gating in a sense of establishing a threshold was never applied, the mean fluorescence signal measured by cps in the ROI was used to calculate the ICC.**

**In addition, we provide images of the ICG clearance in the first hour during the pilot phase of the study (Supplementary Fig 1). This images and assessment were performed with the Fluobeam NIR camera and not the VIS-BIR-Xray imaging since the clearance observations were established during the induction of the first SL animals (pilot group). In the injection site, the ICG remained positive for several hours. The subcutaneous injection sites hold some degree of ICG signal probably because parts of the molecules bind to tissue proteins. However, the site of injection is not relevant for the analysis since the ROI were defined excluding the area of ICG injection. The ICG clearance capacity was always calculated at predefined ROI from the VIS-BIR-Xray image sequence after 1 h. Here, the ICG was not really visible in healthy rats. We hope that this explanation fully meets the reviewer’s concerns, and has improved the readability of the paper.**

3. In addition to a new approach to generating a lymphedema model in the rat, the primary new finding of the manuscript is the presence of this aSMA+/Vimentin+/HSP+ cell type that is present in the skin at later stages. It would add tremendous value to this data if the authors could figure out a way to better capture this rich data set across the different time points and locations, similar as to what was done for Figure 5 for collagen. Could the number of aSMA+/Vimentin+/HSP cells be counted to show their increase over disease progression? Is this localized primarily to the location of the initial lymphatic injury or is it seen in the distal end of the injured limb?

**A: We agree that complementing the images with a clear quantitative approach would add additional impact to the findings. However, we are also aware that during the preparation of the slides some cells might be located so close to each other that they appear to be one, or on the contrary, and depending on the cutting angle two smaller cells might be counted as one. In contrast, the information obtained from the stained collagen slides is technically far more reliable since the whole slides were scanned at fixed settings. This is not technically possible in confocal microscopy because it would take several hours to scan just a single slide and the focus for one region of the slide might generate distorted images at another region.**

**Nevertheless, since images of each slide were obtained before with confocal microscope at the same predefined ROI coordinates in order to establish a standardized and representative pattern for the aSMA+/HSP47+/vimentin+ cells, it is procedurally possible to establish retrospectively a quantitative approximation of this phenotype at different timepoints. Therefore, the already obtained confocal images were randomized and the aSMA+/HSP47+/vimentin+ cells counted by a blinded observed. This cell counts per field of view was then statistically analyzed and compared in the revised version of the manuscript (Fig 8). This additional evidence showed, that zone C (tight) distal to the injury site was the area with more cells. On the other hand, the injection site (foot dorsum) revealed the lowest count of triple positive cells at all timepoints. Such evidence suggests, that the subcutaneous injections of ICG per se are not likely to be the cause for the phenotypical shift of the fibroblasts. We hope, that this additional effort in validating the results is appreciated by the editor and reviewers. Nonetheless, an additional figure was generated for the main body of the paper (Fig 8).**

4. The manuscript would be greatly enhanced by a figure that summarizes the kinetics of the pathological changes in this animal model. A primary strength of the model is the thoroughness with which the authors looked at lymphatic physiology, collagen, lipid, mast cells, and myofibroblasts at all of these different time-points in both the injured limb and the contralateral control. For the histology, this was done in three different zones as well

**A: We think that this idea of Reviever#1 would in fact be a great improvement for the paper since it would work as a sort of “graphic abstract” for the readership more prone to assimilate information by figurative representations. A figure of the summarized outcomes is provided in the revised version (Fig 9). The editor should now decide whether this new figure could be part of the main body or should be a supplementary figure of the paper.**

Minor comments

1. I recommend replacing the phrase “stage migration” with the more descriptive phrase “lymphedema stage progression”. The term “stage migration” has never been used in the lymphedema literature and it is not immediately obvious what exactly this term means. I assume that the authors are using this to refer to the advancement of the pathology through the various clinical lymphedema stages according to the classification stages used by the ISL. While they do define more clearly what they mean by the term on p.9, the term appear in the title, the abstract, and in numerous locations in the early pages of the paper before the reader is told what the term means.

**A: We do agree with the Reviewer#1 that the term might be confusing for parts of the readership and that despite defined in the methods, earlier appearances in the paper do not encourage the overall comprehension. Consequently, and following the suggestion, we changed the term “stage migration” for “lymphedema stage progression /stage progression” in all sections of the manuscript.**

2. “Unreliable in vivo generation of SL” (p. 3), while listed as a weakness by the authors of current models, could just as easily be considered a strength. The authors heavily emphasize the “translatability” of their model as it relates to human lymphedema. Wouldn’t the more translatable model be one were lymphedema incidence is not 100%, as it is nowhere close to this rate in the clinical surgeries associated with lymphedema incidence.

A: **We appreciate the discussion that has been raised and the interesting point of view. An animal model that aims to mimic a pathology in terms of translatability requires to be reproducible and standardized. To this regard it is not possible to agree that instable and unreliable incidence rates of SL would benefit the science.**

**Erratic lymphedema incidence in preclinical models will inevitably have as consequence that for each study cohort more animals would be required for each experimental setting. To get significant or consistent observations in a model intrinsically prone to diversity of responses, the “minimum number to intervene” must be greater.**

**More experimental animals because of problems with the consistency of the SL model violates the “reduce principle” and counterposes the intention of the directive 2010/63/EU of the European Union (and others) governing experimental animal use. A final reason for pursuing consistency on the in-vivo models is that otherwise during preclinical therapeutic stages, a significant cost increment must be expected. In sum, despite that the argument that heterogeneity of responses translatability fits our species better is tempting, however this approach opposes the standard procedures of modern science.**

3. In the abstract I suggest replacing the phrase “translatable tool” with “clinically relevant model” as an model is not translatable in the general use of the work, which is typically reserved for therapies, technologies, etc.. that could be used in the clinic.

**A: The remark of Reviewer#1 is gratefully accepted and the concept changed in the revised manuscript.**

4. Remove the term “solid” from the subtitle at the top of page 13 as this adjective is vague in this context and does not add meaning.

**A: The remark of Reviewer#1 is accepted and the adjective removed in the revised manuscript.**

5. Statistical markings (e.g. * and **) need to be directly on the figures so that the reader can directly determine which comparisons are significant and what the p value range is.

**A: The markings were included to the figures for better visualization**

6. I think it is an overgeneralization to state there is a lack of surgical refinement and intraoperative mapping inherent to the rodent tail (p. 18). While I am sure there is a large variation across the different groups that have used this technique, collecting lymphatics can be easily visualized with methylene blue injection and carefully injured in a manner that avoids unspecific venous insufficiency (for example see the methods section describing the method in Tabibiazaret et al., PLoS Medicine, 2006).

A: **The publication of Tabibiazar describes in the method section the induction in the mice tail model as follows** ***“…the tail was circumferentially incised proximally, at a point 16 mm distal to its base. The major lymphatic trunks were identiﬁed through subcutaneous injection of methylene blue distal to the surgical incision, followed by controlled, limited cautery ablation of these structures.”*** **We could not find a description here of how the lymphatics were easily identified and carefully injured in a manner that avoids unspecific venous insufficiency (as stated by Reviewer#1). However, the postoperative analysis and molecular insight offered in this paper was done masterfully. Nonetheless, based on the described methodology we cannot agree with the reviewer that in this paper methylene blue dye was employed in any distinctive fashion from the rest of the SL tail models described. Therefore, it is in our opinion unlikely that the method of Tabibiazar et al provide additional reliability of the SL induction. However, we can agree that the term “inherent to tail models” is unfortunate since it may be taken as a judgement to all tail models. It was rephrased in the new version to avoid overgeneralization.**

7. Intro, page 3: Identification of lymphatics with dyes such as methylene blue and evan’s blue suffer the same limitations in regard to their lymphatic specificity as does ICG. When injected in small doses into the skin, they have a very high affinity to albumin, which they bind to and are then drained by lymphatics due to the molecular weight of albumin excluding their uptake by the circulation. ICG certainly enhances visualization of lymphatics if one has the appropriate optics, but a trained researcher can easily identify and remove lymphatics with blue dye labeling as well.

**A: Although, a trained researcher might identify and remove lymphatics after injection and labeling of blue dyes, the ICG technique offers clear advantages in terms of transcutaneous visualization, real-time imaging and high sensitivity compared to blue dye alone. In addition, ICG lymphangiography has evolved into a standard visualization technique for clinical lymphedema imaging and sentinel lymph node biopsy replacing completely the use of blue dyes. As the reviewer#1 points out, “blue dyes” like Evans blue shares the same limitations with ICG. We agree that a trained surgeon or researcher might also identify lymphatics with blue dyes or just microscopic visualization AFTER incision what implies limitations in the preoperative planing. Since we agree with the reviewer that ICG is the superior visualization technique but shares some limitations with the alternative dyes, and that training/experience is required to remove lymphatics especially with blue contrast dyes, we do not understand where the critic is to our work. In sum, we all agree that ICG seems to be the better alternative to blue dyes for the imaging of SL models, but blue dyes could still be used if the scientist or surgeons is well trained and experienced.**

8. The next revision need to be carefully read for grammar as there are numerous mistakes throughout the manuscript.

**A: The revised version has been carefully checked for potential mistakes by us and a native speaking college. If there are still mistakes that could endanger the comprehension of the paper, we would be thankful if such could be noted.**

9. This was mentioned in the last revision, but NIR imaging has been used to identify lymphatics and guide surgical injury in animal models in other publications: Weiler, Sci Rep, 2019 and Nelson, Nature Biom Eng, 2019. There is still a statement in the manuscript that NIR has nor been used to “guide a precise lymphatic injury for in vivo studies” (p.3) that is not accurate.

**A: We thank reviewer#1 for the precise management of the literature. Unfortunately, we cannot agree to this opinion. In details:**

a) **Despite an outstanding paper, Nelson 2019 described in the methods “Before the study, three (of note not all) of the sheep underwent gadolinium-enhanced MRI to structurally map the lymphatic anatomy of the hind limbs. 1 d after the MRI, the sheep received the initial NIR functional lymphatic imaging procedure on both hind limbs. After imaging (not while), collecting lymphatic ligation surgery was conducted on one of the hind limbs, with the contralateral limb remaining intact to act as an internal control. …the induction itself…** **exposing a collecting lymphatic vessel running in parallel to the saphenous vein. A 2-cm portion of the vessel along with the vein was ligated with sutures and resected”**
b) **In the methods of Weiler 2019 …”All three groups received incisions 1 cm from the base of the animal spanning 80–90% of the circumference of the tail with particular care to standardize the incisions as much as possible. In the vessel ligation groups, the incision severed one collecting vessel and left one intact, while in the sham control group the incision severed a substantial percentage of the lymphatic capillary network, but none of the collectors were severed. All incisions were cauterized to prevent bleeding and fluid leakage. After the surgical procedures (not before or while proceeding), both collecting vessels were checked with NIR imaging to ensure they were either severed or remained intact as appropriate. Animals in which vessels were improperly ligated or in which blood vessels were ligated were excluded from the study.”**

**We hope that the reviewers agree with us, that the first paper performed lymphatic mapping in a proportion of the animals before the induction with MRI, and not NIR. They used this technology AFTER the surgery, and not for surgical navigation or injury. In fact, they ligated a main vein of the sheep along with the lymphatic collector which is -again - a plausible cause for a concomitant venous insufficiency.**

**The second cited publication performs a standardized excision of most of the rodent tail circumference and tests the animals for lymphatic collector excision after the surgery. By no means it is therefore possible to claim that the precision of a surgical injury could be enhanced by postoperative assessment. Excluding unprecise injury is a quality check of the surgical injury but not a methodological optimization to effectively enhance the injury precision while generating the model. Additionally, this approach excluded animals (not stated how many) with NIR because of unprecise injury, costing unnecessary animal lives by this approach. We on the other hand performed a color-coded NIR lymphatic mapping on all the animals intervened before the surgery and complemented a microscopically assisted visualization with intraoperative real-time NIR for lymphatic injury guidance. By all intent to not “overrate” the SL model proposed, we must underline our technique and the conclusion, that NIR has not been used to guide a precise lymphatic injury.**

10. The authors need to replace references to rat tail in the manuscript with mouse tail as almost all lymphedema models in the tail have been done in mice. There are no good rat tail models of lymphedema due to the large amount of cartilage (as mentioned by the author), however this is not true of the mouse tail and the histological changes in the mouse tail are remarkably similar to the human and to those reported in the paper here.

**A: Considering that we proposed a rat model, our primary intention was to compare and discuss about model’s analogue to our model. Yet, we agree that there is more literature of SL mice tail models than studies on rat tails. In order to not overrepresent the rat proportion of the tail models and give a false literature impression to the reader we agreed to replace some references in the revised version for mice tail models.**

11. I suggest the authors downplay how translational their model is. As mentioned before every animal undergoing surgery swell above a “lymphedema threshold”, the appearance of lymphedema occurs on the first day after surgery, and every animal’s swelling appears to be resolving on its own without any intervention. None of these three features are common in clinical lymphedema. This model is definitely valuable, but it seems to suffer the most crucial limitations of all current rodent models of lymphedema.

**A: We appreciate the reviewer’s ongoing discussion: From our perspective the model has true merit to overcome several pre-published limitations and might serve as a valuable translational model. In order satisfy the reviewer#1, we have slightly put the revised version into a modified perspective.**

**Overall, we specially thank Reviewer#1 for taking the time and effort to suggest improvements and detailed analysis of our work. Also, in this review he/she was able to recognize the strength and improvements of our paper. Such a commitment to the job is exemplary and seldomly seen. Despite the fact that the corrections required a significant additional effort, we realized that the final manuscript now has gained significant benefit based on the reviewers´efforts. Thank you.**

------------------------------------------------------------------------------------------------------------------------------------------------------------

Reviewer #2: The authors have satisfactorily addressed the comments by the previous reviewers

**A: We thank Reviewer#2 for the positive feedback and hope that this manuscript version meets all the criteria of PLOS One and the expectations of the editor and reviewers.**
